# Global prevalence of E-cigarette use among students: Systematic review and meta-analysis

**DOI:** 10.1371/journal.pone.0332160

**Published:** 2025-12-01

**Authors:** Natnael Atnafu Gebeyehu, Kelemu Abebe Gelaw, Yibeltal Assefa Atalay, Belete Gelaw Walle, Molalegn Mesele Gesese, Biruk Adie Admass, Belete Birhan, Awoke Elefachew Geberemariam, Biresaw Wassihun Alemu, Nathan Estifanos Shewangashaw, Kirubel Dagnaw Tegegne

**Affiliations:** 1 School of Midwifery, College of Health Science and Medicine, Wolaita Sodo University, Wolaita Sodo, Ethiopia; 2 Department of Public Health, College of Medicine and Health Science, Wolaita Sodo University, Wolaita Sodo, Ethiopia; 3 Department pediatrics and Neonatal Nursing, School of Nursing, Wolaita Sodo University, Wolaita Sodo, Ethiopia; 4 Department of Anesthesia, College of Medicine and Health Sciences, University of Gondar, Gondar, Ethiopia; 5 Department of Psychiatry, College of Health Science and Medicine, Wolaita Sodo University, Wolaita Sodo, Ethiopia; 6 Department of Nursing, College of Medicine and Health Sciences, Debre Berhan University, Debre Berhan, Ethiopia; 7 Department of Midwifery, College of Medicine and Health Sciences, Injibara University, Injibara, Ethiopia; 8 Department of Adult Health Nursing, College of Medicine and Health Sciences, Wollo University, Dessie, Ethiopia; University of Saskatchewan, CANADA

## Abstract

**Background:**

The growing use of e-cigarettes among students is a major public health concern. Yet, global data on its prevalence and associated risk factors remain limited. Therefore, this study aimed to estimate the global prevalence of e-cigarette use among students and identify key predictors influencing usage patterns.

**Methods:**

A systematic review and meta-analysis were conducted using articles retrieved from databases including Science Direct, Scopus, EMBASE, Google Scholar, and PubMed, between August 15 and September 21, 2024. Data were extracted using Excel and analyzed with STATA version 14. Heterogeneity was assessed using the I^2^ statistic, and publication bias was evaluated through forest plots and Begg and Egger’s tests. Subgroup analyses were conducted by geographic region, World Bank income classification, and level of education. A pooled odds ratio was calculated to identify predictors of e-cigarette use.

**Results:**

A total of 40 studies with 654,853 student participants were included in the final analysis. The global prevalence of e-cigarette use among students was 22.65%. Usage varied significantly by region and demographic factors, with the highest rates observed in the Western Pacific (32.13%) and among high school students (33.62%). Students in high-income countries reported the highest usage (23.15%) as well. Key predictors of e-cigarette use included being male (AOR = 3.22), smoking conventional cigarettes (AOR = 5.35), and consuming alcohol (AOR = 3.14).

**Conclusion:**

This meta-analysis reveals a high global prevalence of e-cigarette use among students, especially among high school males in high-income and Western Pacific regions, with strong associations to conventional cigarette smoking and alcohol use. Prioritize region-specific school based interventions targeting high-risk students particularly high school males in high-income countries to curb e-cigarette use and associated behaviors like smoking conventional cigarettes and alcohol consumption.

## Introduction

Electronic cigarettes are battery-powered gadgets that produce an aerosol due to the heating of a solution generally flavored, humectant, and having nicotine. Electronic smoking or e-smoking is characterized as inhalation of the aerosol [1,2]. Consumption of e-cigarettes is currently increasing everywhere globally, especially among youths [3]. According to 2021 Global burden of disease, tobacco remains a health risk globally (at 5.7% of all DALYs) is third after air pollution and blood pressure [4]. The countries are now taking measures over harm reduction after consumption of tobacco. Because adolescent smoking usually starts with the majority of smokers at adolescence time [5], preventing adolescent smoking quitting and transition towards established smoking are two important yet poorly represented subjects in public health.

E-cigarettes were the second most used nicotine product in the United States in 2020–2021, according to the National Center for Chronic Disease Prevention and Health Promotion [6]. In the European Union in 2012, only 7.2% of adults over 15 reported having ever tried an e-cigarette; meanwhile, such a phenomenon was much more plausible among students at the same time [7]. In a study conducted on students of three universities in the United Arab Emirates, it was established that 23% of participants smoked e-cigarettes across the Arab world [8].

The factors influencing e-cigarette use among college-aged young adults have led to an astonishing rise in consumption [9–14]. This has been due to the misleading notion that the usage of e-cigarettes is less hazardous than traditional cigarettes and can help users quit smoking [15–17]. Other contributors include easy availability, increased prevalence, peer pressure, and curiosity [18].

Research findings indicate that e-cigarettes influence the symptoms associated with cigarette withdrawal [19]. Many other studies reported young people who used the e-cigarette are more likely to smoke conventional cigarettes [20]. The use of these devices poses a great public health risk due to either current smokers continuing with the habit or nonsmokers getting hooked on nicotine [21,22]. E-cigarette consumption increases mucus secretion, causes lung damage, cilia dysfunction, and airway inflammation [23,24]. Acute and chronic health disorders such as myocardial infarction, coronary heart disease, stroke and arteriosclerosis occur more frequently in e-cigarette users than in non-users [25–27].

It constitutes a disruptive change that complicates tobacco control worldwide [28–30]. Because of these concerns, countries are issuing various public health policies on the use of e-cigarettes [31]. Although the relative benefits and harms of e-cigarettes to long-term users are still debated [32], the toxicological profile of e-cigarettes and the specific effects of nicotine on adolescents, in particular, college students, constitute a unique concern [33,34]. Besides the direct harms of e-cigarettes, one of the key concerns with this vulnerable population is that it may lead to the use of tobacco cigarettes and associated lifelong problems, undermining tobacco control efforts. Evidence is needed to guide public health policy in this important area [35].

There is substantial heterogeneity in global studies reporting the prevalence of e-cigarette use among students and the factors associated with it, with rates ranging from as low as 0.9% in high-income countries to as high as 75% in low-income countries. These wide disparities reflect differences in cultural, regulatory, socioeconomic, behavioral, and methodological contexts. However, the absence of a comprehensive synthesis of these varied findings limits our understanding and hinders the development of effective, context-specific interventions. This study aims to fill that gap by systematically pooling global data and identifying associated factors to provide robust evidence that can inform tailored tobacco control policies and enhance youth prevention efforts worldwide.

## Methods

### Reporting

The current review has strictly followed the checklist of PRISMA (Preferred Reporting Items for Systematic Reviews and Meta-Analysis) guidelines [36] (S1 File). In addition, the protocol for this systematic review and meta-analysis has been registered in the International Prospective Register of Systematic Reviews (PROSPERO) under registration number CRD42024606377.

### Search strategy

Comprehensive literature search was conducted on Pub Med, Science Direct, Scopus, EMBASE, and Google Scholar. The search was performed from August 15, 2024, to September 21, 2024, with different terms and keywords such as “prevalence,” “electronic cigarette use,” “e-cigarette use,” “determinants,” “factors,” “predictors,” and “students.” These keywords were searched individually and in combination together using Boolean operators “OR” and “AND.” databases used to fetch the research articles. The PubMed search strategy used was: ((((“epidemiology”[MeSH Subheading] OR “prevalence”[MeSH Terms] OR Prevalence [Text Word]) AND (“electronic nicotine delivery systems”[MeSH Terms] OR E-cigarette [Text Word]))) AND (factors [All Fields])). We used PECO guide (Population, Exposure, Comparator and Outcome) format for the explicit articulation of our review question and explicit clarification of inclusion and exclusion criteria. Since our included studies are observation, We refrained using PECO guide for confirmation.

**Main review questions:**

1. What is the global prevalence of e-cigarette use among students?
2. What factors are associated with e-cigarette use among students globally?

### PECO guide

**P (Population):** Students (could be secondary, high school, or university students).

**E (Exposure):** Use of e-cigarettes.

**C (Comparator):** Not applicable or students who don’t use e-cigarettes.

**O (Outcome):** Prevalence of e-cigarette use.

### Inclusion and exclusion criteria

The systematic review and meta-analysis included articles that contained the following criteria: 1. Type of study: All observational studies that documented the prevalence of the use of electronic cigarettes; 2. Population: Students; 3. Language: English; 4. Context: Global; 5. Full text availability at the time of search. It is important to note that this systematic review and meta-analysis excluded qualitative studies, those for which full texts were unavailable, letters to the editor, review articles, expert opinions, case studies, case series, and randomized controlled trials.

### Outcome measurement

The term “ever use e-cigarettes” was operationalized by a positive response to the query: “Have you ever tried or experimented with electronic cigarettes, e-cigarettes, or e-cigarettes, even if just for one or two puffs?” The available answer options were “yes” and “no” [37]. On the other hand, the “current e-cigarette use” was established by the “yes” answer to the question: “Have you used an electronic cigarette in the last 30 days?” [38].

### Quality assessment

This study used a standardized quality assessment checklist from the Joanna Briggs Institute [39] to assess the quality of the reviewed studies. Two authors, NAG and KDT, independently reviewed the studies. The critical appraisal checklist The eight items check list for critical appraisal with “Yes,” “No”, “Uncertain”, and “Not Applicable” response for each item was as follows: explicit inclusion criteria for the sample stated; description of study participants and setting provided; exposure measured in a valid and reliable way; primary objectives and predefined standards achieved; potential confounding variables reported; actual measure reported for confounding factors; measurements of outcome variable precise; appropriate statistical analysis applied. All discrepancies in the quality assessment process were resolved through discussion and by a mutual agreement by the third author, KAG. Any study that scored 50% and above in the quality assessment process was considered as having a low risk. To ascertain inter-rater reliability at the full-text screening stage, Cohen’s kappa statistic was applied. Kappa was 0.84, indicating nearly perfect agreement between the two raters. According to Landis and Koch’s benchmark scale, kappa of 0 indicates poor agreement; 0.01–0.20, slight agreement; 0.21–0.40, fair agreement; 0.41–0.60, moderate agreement; 0.61–0.80, substantial agreement; and 0.81–1.00, nearly perfect agreement.

### Data extraction

Data extraction and analysis were conducted using a Microsoft Excel spreadsheet from 2016 and STATA (version 14) software respectively. To ensure consistency and accuracy, a standardized data extraction format from the Joanna Briggs Institute was utilized by two authors (NAG and KDT), who independently collected all relevant data. Any discrepancies that arose during the extraction process were resolved through discussions led by the third author (YAA), resulting in a consensus among the authors. The extracted data included the first author’s name, publication year, country of study, study setting, study design, sample size, prevalence of e-cigarette use, and the quality assessment of each paper.

### Data analysis

Data from a Microsoft Excel spreadsheet (2016) were imported to STATA software version 14 for the analysis. Generally, in data pooling, there are two ways to do it: the two-step method and the one-step method. The two-step method includes the preliminary stage of data cleaning and then applies a uniform or commonly accepted cut-off value for each scale. This approach categorizes the status of each participant as either ‘yes’ or ‘no’ in assessing the use of electronic cigarettes, then calculates the prevalence based on the study population. The two-step approach is the most commonly used to pool prevalence data from several studies. It involved abstracting the overall percentage of e-cigarette use from each of the studies included and then conducting a synthesis using a random-effects model on the prevalence estimates via STATA statistical software. Subgroup analyses were also conducted according to study region, study population, and income category. Furthermore, subgroup analyses were conducted for each of the WHO regions, World Bank Regions, and World Bank income categories. The influence of any single study on the overall meta-analysis estimate of prevalence was investigated by a sensitivity analysis. Publication bias was investigated through constructing a funnel plot and conducting regression tests by Begg and Egger for more objective analyses. Heterogeneity was tested and the total/residual heterogeneity estimated using Cochran’s Q X^2^ test and I^2^ statistics, respectively [40]. In addition, univariate meta-regression analyses were performed to assess the impact of both sample size variation and publication year variation on the heterogeneity observed among studies [40].

### Ethics approval and consent to participants

Not applicable because no primary data were collected.

### Meta-analysis

**Fig 1:**
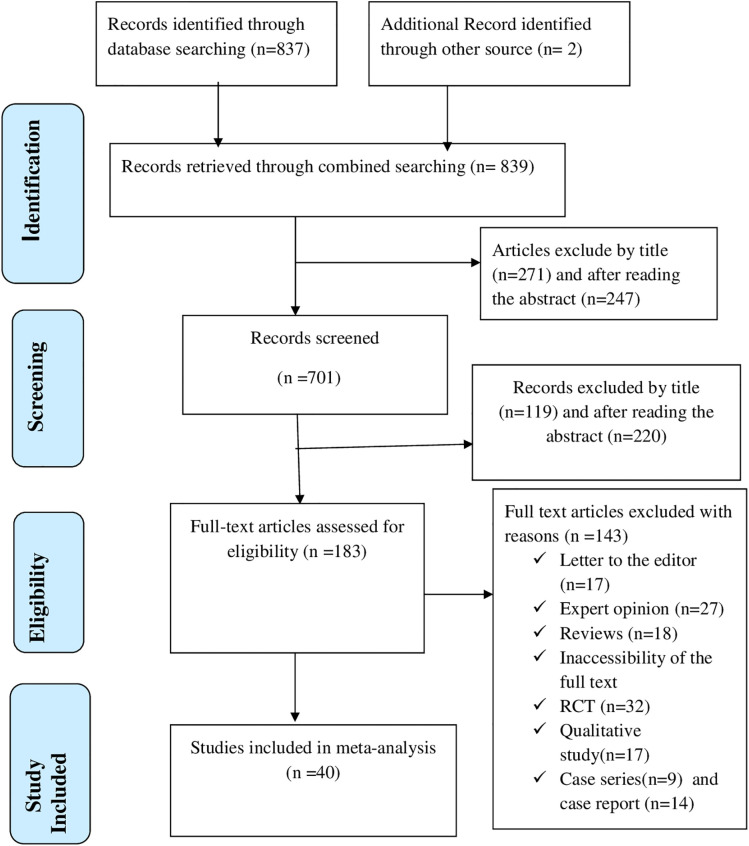
A Prisma diagrammatic presentation used to show the selection of studies. The inclusion criteria were all observational studies reported e-cigarette prevalence, student participants, published in English studied globally, and availability of full text. Studies were excluded if they were qualitative studies, letters to the editor, review articles, expert opinions, case studies, case series, and randomized controlled trials.

Our research began with a comprehensive search strategy across various global electronic databases to identify 839 studies. After removal of 138 duplicate entries, a total of 701 studies underwent a thorough title and abstract review, following which 518 were excluded. After re-screening the full texts of the remaining 183, for various reasons, 143 were excluded: qualitative study; not full text available; letter to the editor, review, expert opinion, case study, case series, RCT. After all, a total of 40 studies [37,38,41–78] with 654,853 participants were identified to meet the inclusion criteria for this systematic review and meta-analysis (Fig 1).

All the reviewed studies were cross-sectional and in institutional settings. Geographically, the studies came from: six USA [45,47,52,53,65,78]; four Saudi Arabia [49,50,56,67]; two Poland [41,43]; two China [44,54]; two France [51,71]; two Palestine [60,62]; two Malaysia [61,72]; two Indonesia [46,73]; and two Canada [48,75]. There was also one study each from the Philippines [37], Greece [38], Austria [42], Slovakia [55], Pakistan [57], Serbia [58], Italy [59], Vietnam [63], Jordan [64], Germany and Hungary [66], Brazil [68], Qatar [69], New Zealand [70], Ecuador [74], Iraq [76], and Thailand [77]. Of the overall studies, twenty-five reported on university students, nine on high school students, while two involved all types of students. Eighteen studies described lifetime e-cigarette use. Ever and current use were evaluated in 16 studies; six studies described only current use. The highest reported prevalence of ever use of e-cigarettes was 26.9%, and the lowest, 4.9%. Sample sizes for the studies varied from 441,900 participants to 401 participants. Based on the analysis, all studies had a low risk of bias (**Table 1**).

**Table 1:** Characteristics of studies included in the systematic review and meta-analysis of global prevalence of electronic cigarette use among students.

Author/year	Country	Study setting	Study design	Study population	Sample size	Ever use	Current use	Quality
Duplaga M and Grysztar M/2022 [41]	Poland	Institution	Cross-sectional	High school	2223	47.5	18.6	Low risk
Leung J. et.al/2023	Australia	Institution	Cross-sectional	High school	855	74	NR	Low risk
Serra, C/2021 [37]	Philippines	Institution	Cross-sectional	High school	6670	24.6	14.1	Low risk
Janik- K. et.al/2020 [43]	Poland	Institution	Cross-sectional	High school	5154	NR	26.9	Low risk
Chen J. et.al/2019 [44]	China	Institution	Cross-sectional	Secondary school	40202	5.3	4	Low risk
Soteriades, S et.al/2020 [38]	Greece	Institution	Cross-sectional	High school	4618	12.3	2.8	Low risk
Huang LL. et.al/2016 [45]	USA	Institution	Cross-sectional	High school	4092	NR	7.7	Low risk
Bigwanto et al/2019 [46]	Indonesia	Institution	Cross-sectional	High school	767	32.2	11.8	Low risk
E. Westling et al/2017 [47]	USA	Institution	Cross-sectional	High school	1091	27.7	16.8	Low risk
S. Azagba, et al/2019 [48]	Canada	Institution	Cross-sectional	Secondary school	51661	NR	55	Low risk
Qanash, et al/2018 [49]	Saudi	Institution	Cross-sectional	University	1007	27.7	NR	Low risk
Alzahrani T/2023 [50]	Saudi	Institution	Cross-sectional	University	519	24	NR	Low risk
Tavolacci M-P/2016 [51]	France	Institution	Cross-sectional	University	1134	23	25.3	Low risk
A.M. Franks et al/2017 [52]	USA	Institution	Cross-sectional	University	853	24.2	NR	Low risk
E.L. Sutfin et al/2013 [53]	USA	Institution	Cross-sectional	University	4444	4.9	1.5	Low risk
Song et al/2023 [54]	China	Institution	Cross-sectional	University	9361	16.7	NR	Low risk
Babjakova, J/ 2020 [55]	Slovakia	Institution	Cross-sectional	University	577	19.3	14	Low risk
Alzalabani/2020 [56]	Saudi	Institution	Cross-sectional	University	527	15.9	NR	Low risk
Iqbal et al/2018 [57]	Pakistan	Institution	Cross-sectional	University	500	6.2	NR	Low risk
Irena Ilic et.al/2019 [58]	Serbia	Institution	Cross-sectional	University	404	9.9	NR	Low risk
Canzan et al./2019 [59]	Italy	Institution	Cross-sectional	University	1463	30.3	2.1	Low risk
Ghanim M/2024 [60]	Palestine	Institution	Cross-sectional	University	1002	18.1	NR	Low risk
Puteh SE et.al/2018 [61]	Malaysia	Institution	Cross-sectional	University	1302	74.9	NR	Low risk
Nazzal Z, et al/2024 [62]	Palestine	Institution	Cross-sectional	University	1792	19.7	NR	Low risk
Thanh Huong et al/2022 [63]	Vietnam	Institution	Cross-sectional	University	554	7.2	13.2	Low risk
Al-Sawalha et al/2021 [64]	Jordan	Institution	Cross-sectional	University	1259	11	NR	Low risk
D. R. Kenne et al/2015 [65]	USA	Institution	Cross-sectional	University	9077	27.9	16.3	Low risk
Balogh et al/2018 [66]	German & Hungary	Institution	Cross-sectional	University	2925	0.9	NR	Low risk
Habib, et al/2020 [67]	Saudi	Institution	Cross-sectional	University	401	12.2	NR	Low risk
Oliveira WJC/2017 [68]	Brazil	Institution	Cross-sectional	University	489	NR	0.6	Low risk
Kurdi et al/2021 [69]	Qatar	Institution	Cross-sectional	University	199	14	NR	Low risk
Wamamili B, et al/2020 [70]	New Zealand	Institution	Cross-sectional	University	1476	40.5	6.1	Low risk
Kinouani S/2017 [71]	France	Institution	Cross-sectional	University	2712	NR	3.6	Low risk
Jane Ling et al/2022 [72]	Malaysia	Institution	Cross-sectional	Secondary school	22228	NR	9.1	Low risk
Susi Ari Kristina et al/2020 [73]	Indonesia	Institution	Cross-sectional	All students	920	10.68	NR	Low risk
Ivan Cherrez-Ojeda/2024 [74]	Ecuador	Institution	Cross-sectional	All students	3608	21	16	Low risk
Annie Montreuil et.al/2017 [75]	Canada	Institution	Cross-sectional	High school	441900	17.7	5.7	Low risk
Muhammad Ahmed S/ 2024 [76]	Iraq	Institution	Cross-sectional	University	629	15.7	NR	Low risk
Wichaidit W et.al/2023 [77]	Thailand	Institution	Cross-sectional	Secondary school	23659	12.3	NR	Low risk
Andrew K et.al/2015 [78]	USA	Institution	Cross-sectional	University	599	21	14	Low risk

### Global prevalence of electronic cigarette use

**Fig 2:**
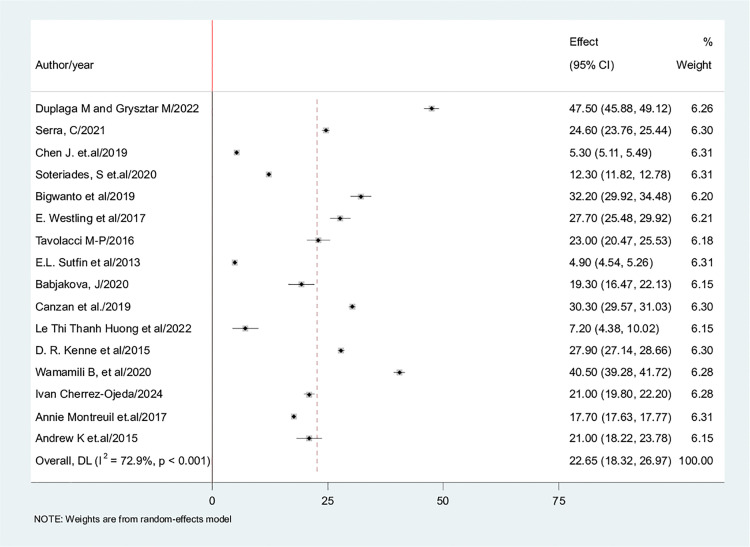
Forest plot of global prevalence of e-cigarette with the height of the diamond is the overall effect size (22.65% while the width is the confidence interval at (95% CI: 18.32, 26.92),The y-axis shows the standard error of each study while the x-axis the estimate of effect size of the each study. The verticalline denotes the no effect. The box represents the effect size of each study and the line across the box is confidence interval of each study.

The current study applied the random-effects model to arrive at the pooled estimate of electronic cigarette use among students. The results indicated that the worldwide prevalence of e-cigarette ever use was estimated at 22.65% [95% CI: 18.32, 26.92], I^2 ^= 72.9% (Fig 2) and current use of 12.95% [95% CI: 8.25, 17.66], I^2^ = 65%.

### Subgroup analysis

The subgroup analysis was done based on the six WHO regions, World Bank regions, World Bank income category, and study population.

#### The prevalence of electronic cigarette use based WHO regions.

A Therefore, given the high level of heterogeneity present in the studies, a subgroup analysis was carried out based on WHO regions. The results also indicated that the highest prevalence of ever use of E-cigarettes was seen to be in the Western Pacific region: 32.13% (95% CI: 18.99, 45.27), I^2^ = 79%, whereas the lowest prevalence was noted in the Eastern Mediterranean region: 14.43% (95% CI: 12.38, 20.48) I^2^ = 65% (**Table 2**).

**Table 2:** The pooled prevalence of electronic cigarette use among students worldwide 95%CI and heterogeneity estimate with a p-value for sub-group analysis.

Characters	Variables	Pooled estimate	I^2^(p-value)
**WHO Regions**	E-cigarette use		
European region	Ever use	20.45% (8.89–32.08)	71% (0.001)
	Current use	13.28% (6.84–19.72)	43% (0.001)
Western Pacific region	Ever use	32.13% (18.99–45.27)	79% (0.002)
	Current use	8.06% (1.33–14.80)	31% (0.001)
Region of the America	Ever use	20.58% (14.67–26.49)	68% (0.001)
	Current use	14.84% (2.43–27.26)	45% (0.001)
Eastern Mediterranean region	Ever use	14.43% (12.38–20.48)	65% (0.001)
	Current use	0.000%9	0.000%
Southeast Asian region	Ever use	27.44% (7.22–47.66)	82% (0.001)
	Current use	11.10% (8.41–13.80)	29% (0.001)
**World Bank Regions**			
European and Central Asia	Ever use	20.45% (8.89–32.00)	64% (.001)
	Current use	13.28% (6.84–19.72)	43% (0.001)
East Asia and Pacific	Ever use	29.75% (22.21–37.29)	58% (0.001)
	Current use	9.64% (6.17–13.10)	37% (0.001)
North America	Ever use	20.51% (13.75–27.28)	53% (0.001)
	Current use	16.72% (1.61–31.82)	51% (0.001)
Middle East and North Africa	Ever use	17.60% (13.94–21.56)	48% (0.001)
	Current use	0.000%	0.000%
South Asia	Ever use	6.2% (4.09–8.31)	0.00% (0.001)
	Current use	0.000%	0.000%
Latin America and Caribbean	Ever use	21% (19.67–22.33)	0.00% (0.001)
	Current use	8.29% (6.80–23.38)	41% (0.001)
**World Bank-Income category**			
Lower middle income	Ever use	20.31% (13.48–27.14)	51% (0.001)
	Current use	13.35% (11.92–14.78)	60% (0.001)
Upper middle income	Ever use	20.20% (9.50–30.91)	48% (0.001)
	Current use	8.55% (1.47–15.63)	54% (0.001)
High income	Ever use	23.15% (18.98–27.32)	57% (0.001)
	Current use	13.76% (7.79–19.74)	62% (0.001)
**Study population category**			
High school students	Ever use	33.62% (25.08–42.16)	55% (0.001)
	Current	13.01% (9.08–16.93)	51% (0.001)
Secondary school students	Ever use	8.80% (1.94–15.66)	33% (0.001)
	Current use	22.7% (5.57–50.97)	68% (0.001)
University students	Ever use	20.22% (14.45–25.99)	45% (0.001)
	Current	9.54% (5.74–13.35)	46% (0.001)
All type of students	Ever use	15.87% (5.75–25.98)	37% (0.001)
	Current	16% (14.08–17.20)	54% (0.001)

#### The prevalence of electronic cigarette use based on World Bank regions.

The current study seeks to identify the prevalence of e-cigarette use in various World Bank regions. The East Asia and Pacific region exhibited the highest prevalence rate, 29.75% [95% CI: 22.21–37.29], with an I^2^ of 58%, while the lowest prevalence rate was found in the South Asia region, with a prevalence rate of 6.2% [95% CI: 4.09–8.31]; with the I^2^ value equaled 0.00% (**Table 2**).

#### The prevalence of electronic cigarette use based on World Bank income category.

We conducted a subgroup analysis based on the assessment made by the World Bank on national incomes. Thus, high-income countries reported a higher prevalence of electronic cigarette use at 23.15% (95% CI: 18.98–27.32) with an I^2^ value of 57%, while that of the upper-middle-income countries was the lowest at 20.20% (95% CI: 9.50–30.91) with an I^2^ value of 48% (**Table 2**).

#### The prevalence electronic cigarette use based on study population.

There were significant differences in the prevalence of electronic cigarette use by different educational levels. Interestingly, the highest usage rate was among high school students at 33.62% (95% CI: 25.08–42.16), while the I^2^ value was 55%. The rate of electronic cigarette use in secondary school students was as low as 8.8% with 95% CI: 1.94–15.66, where the I^2^ value was 33% (**Table 2**).

### Heterogeneity and publication bias

We performed subgroup analyses in order to develop our conclusion by addressing the regions, income categories, and study populations that contribute to the observed heterogeneity of the study (I^2^ = 72.9%). We further conduct a univariate meta-regression analysis by taking sample size, publication year, continent/region, and study population as covariates to identify the main sources of heterogeneity. The result showed that continent (p = 0.012) and sample size (p = 0.000) have a statistically significant contribution to the variation across the studies (**Table 3**).

**Table 3:** Meta-regression analysis of factors affecting between-study heterogeneity.

Heterogeneity source	Coefficient’s	Standard error	p-value
Year	11.07025	31.6252	0.328
Sample size	13.23113	3.031877	0.000
Continent	4.036366	1.63221	0.012
Study population	1.513002	1.619523	0.327

**Fig 3:**
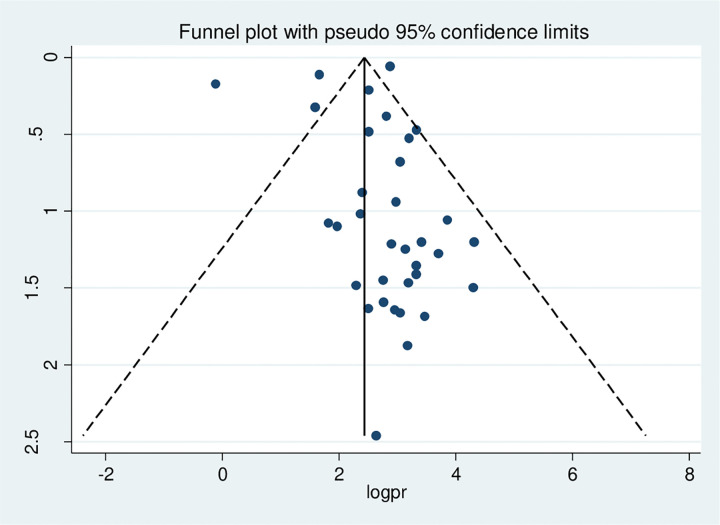
Funnel plot showing asymmetrical distribution of studies indicating the presence of publication bias. The Y-axis is thestandard error and the X-axis is the study result or effect size. The dotted diagonal lie of the funnel is the 95% confidence interval and the vertical. The vertical line is the line of no-effect and dots are included studies reporting electronic cigarette use.

**Fig 4:**
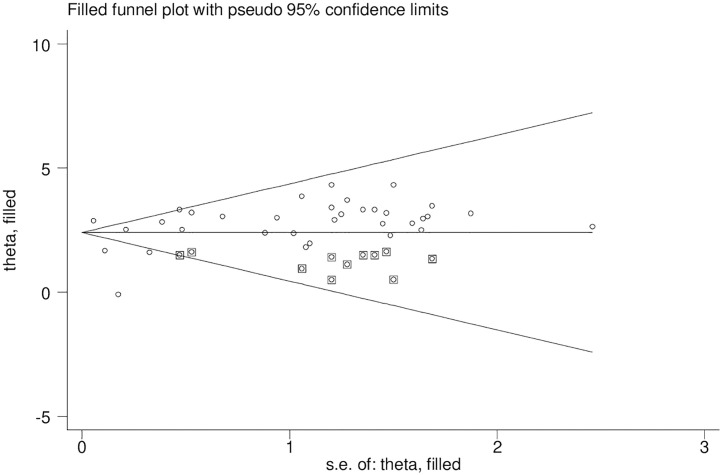
The funnel plot for trim-and-fill method was used to correct the result eleven potential missing studies were requiredin the left side of the funnel plot to ensure symmetry. The enclosed circles represent the dummy studies and the free circles are genuine studies.

**Fig 5:**
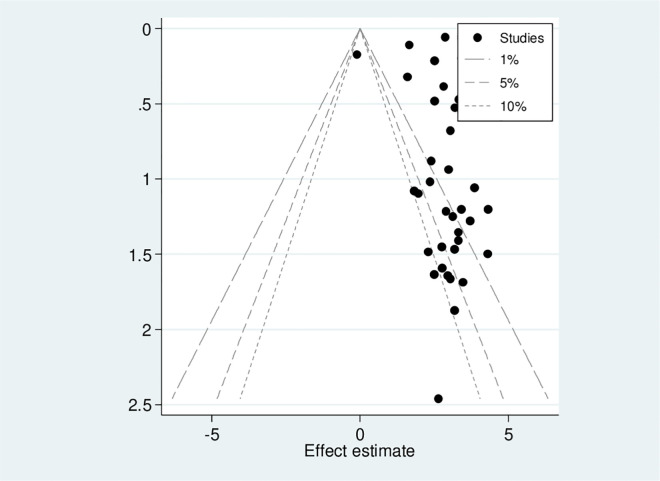
Counter-enhanced funnel plot suggestions of missing studies on the bottom left-hand-side of the plot. Since the majority of this area contains regions of high statistical significance (P < 0.01), this reduces the plausibility that publication bias is the underlying cause of this funnel asymmetry. Various shaded regions indicate statistical significance. In particular, thewhite shaded region in the middle corresponds to p-values greater than.10, the dark gray-shaded region corresponds to p-values between.10 and.05, the medium gray-shaded region corresponds to p-values between.05 and.01, and the region outside of the funnel corresponds to p-values below.01.

Publication bias was assessed with subjective and objective methods in this study, including funnel plot visualization and Egger’s and Begg’s tests. The funnel plot in (Fig 3) shows an asymmetrical distribution among visual observation studies. In addition, results from Begg’s correlation test and Egger’s regression test had p-values of 0.003 and 0.000, respectively, indicating significant publication bias. To address this issue, a Duval and Tweedie trim-and-fill analysis which imputed eleven studies to correct the asymmetry found in the funnel plot, shown in (Fig 4). A counter-enhanced funnel plot analysis was also conducted to further identify the asymmetry present. The findings from this analysis, presented in (Fig 5), imply that publication bias is likely responsible for the observed asymmetry, as the majority of studies are situated within the significant zone.

### Leave –one-out-sensitivity analysis

A leave-one-out sensitivity analysis was undertaken to examine the individual influence of each study on the estimated overall prevalence of electronic cigarette use. This sensitivity analysis showed that removing any single study did not significantly alter the overall global prevalence of e-cigarette use (**Table 4**).

**Table 4:** The pooled prevalence of electronic cigarette use among students globally when one study omitted from the analysis a step at a time.

Author/year	Estimate	(95% Conf.)
Duplaga M and Grysztar M/2022	21.26	18.076–24.439
Leung J. et.al/2023	20.47	17.33–23.61
Serra, C/2021	21.96	18.71–25.20
Janik-Koncewicz K. et.al/2020	22.03	18.84–25.23
Chen J. et.al/2019	22.55	19.16–25.95
Soteriades, S et.al/2020	22.33	19.06–25.61
Huang LL. et.al/2016	22.03	18.84–25.23
Bigwanto et al/2019	21.73	18.50–24.97
E. Westling et al/2017	21.86	18.63–25.10
S. Azagba, et al/2019	22.03	18.84–25.23
Qanash, et al/2018	21.86	18.63–25.10
Alzahrani T/2023	21.98	18.74–25.21
Tavolacci M-P/2016	22.01	18.76–25.25
A.M. Franks et al/2017	21.97	18.73–25.21
E.L. Sutfin et al/2013	22.56	19.30–25.82
Song et al/2023	22.20	18.91–25.49
Babjakova, J/2020	22.12	18.88–25.36
Alzalabani and Eltaher/2020	22.22	18.98–25.46
Iqbal et al/2018	22.52	19.27–25.76
Irena Ilic et.al/2019	22.40	19.16–25.64
Canzan et al./2019	21.78	18.55–25.02
Ghanim M/2024	22.15	18.91–25.40
Puteh SE et.al/2018	20.43	17.35–23.50
Nazzal Z, et al/2024	22.11	18.86–25.35
Le Thi Thanh Huong et al/2022	22.49	19.24–25.73
Al-Sawalha et al/2021	22.37	19.12–25.62
D. R. Kenne et al/2015	21.85	18.63–25.08
Balogh et al/2018	22.68	19.63–25.74
Habib, et al/2020	22.33	19.09–25.57
Oliveira WJC/2017	22.03	18.84–25.23
Kurdi et al/2021	22.27	19.03–25.50
Wamamili B, et al/2020	22.03	18.84–25.23
Kinouani S/2017	22.03	18.84–25.23
Jane Ling et al/2022	22.03	18.84–25.23
Susi Ari Kristina et al/2020	22.38	19.13–25.63
Ivan Cherrez-Ojeda/2024	22.18	18.53–25.82
Annie Montreuil et.al/2017	22.18	18.53–25.82
Muhammad Ahmed S/ 2024	22.23	18.98–25.47
Wichaidit W et.al/2023	22.34	21.91–25.77
Andrew K et.al/2015	22.07	18.83–25.31
Combined	22.03	18.84–25.23

### Factors associated with electronic cigarette use among students

This study examined the factors that may predict electronic cigarette use among students. It considered the variables of gender, use of traditional cigarettes, and current alcohol consumption. The findings indicated that being male, convenient cigarette use, and current alcohol consumption proved to be a statistically significant predictor in the use of electronic cigarettes among respondents.

#### Being male.

**Fig 6:**
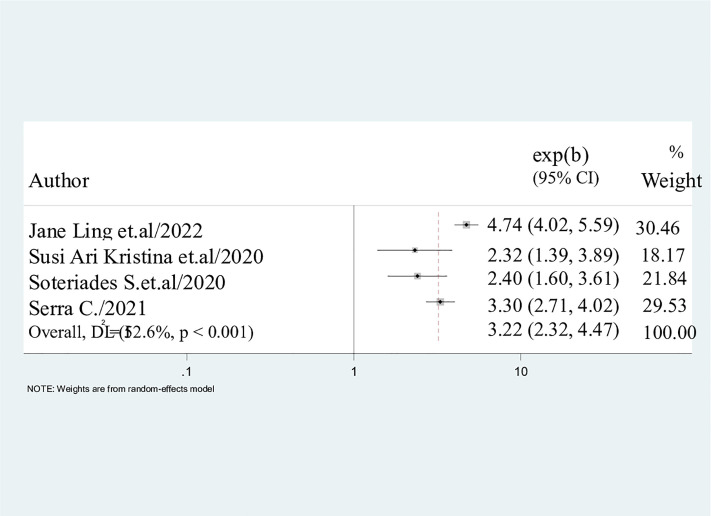
Pooled odds ratio displaying the association between being male and electronic cigarette use. Males were 3.22 times using electronic cigarette smoking than females 95%CI: (2.32, 4.47).

The outcome of this study indicated that male gender was a significant predictor of the use of electronic cigarettes among students worldwide. The results therefore indicated that consumption of the electronic cigarettes was likely to occur three times more in males than females (AOR = 3.22; 95%CI: 2.32, 4.47) (Fig 6).

#### Consumption of convenient cigarette.

**Fig 7:**
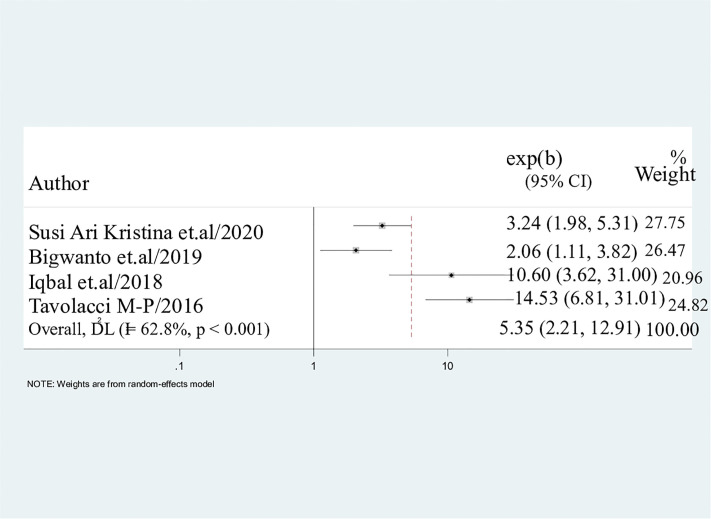
Pooled odds ratio displaying the association between convenient cigarette use and e-cigarette use. Students who utilized convient cigarette use were 5 times more likely to use electronic cigarette 5.35 (2.21, 12.91).

In our research, the use of convenient cigarettes was found to have a significant correlation with the use of electronic cigarettes among students. Consequently, students who engaged in convenient cigarette consumption were four times more likely to utilize electronic cigarettes compared to their peers (AOR = 5.35; 95%CI: 2.21, 12.91) (Fig 7).

#### Current alcohol consumption.

**Fig 8:**
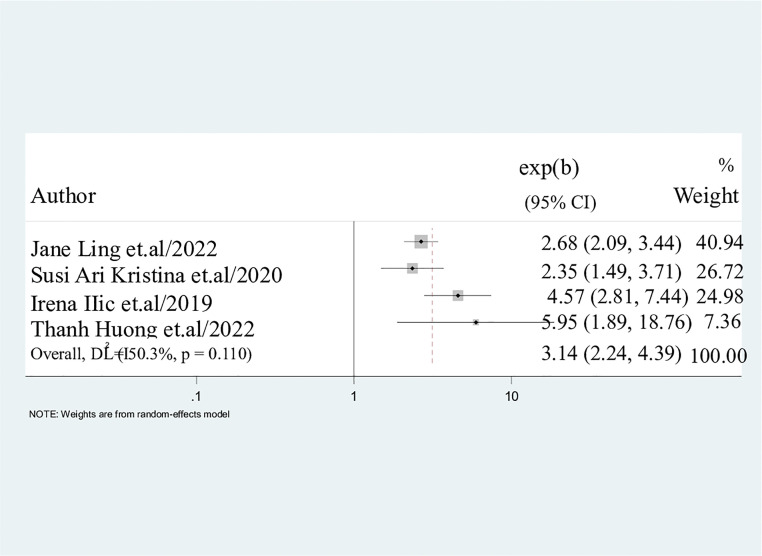
Pooled odds ratio displaying the association between current alcohol consumption and electronic cigarette use. Study participants who used alcohol were 3 times more likely to use electronic cigarette as compared to those who didn’t use 3.14 (2.24, 4.39).

It was observed in the result of this present study that current alcohol consumption significantly predicted the use of electronic cigarettes among the students. As a matter of fact, the findings showed that students who were currently consuming alcohol were four times more likely to use electronic cigarettes than their colleagues who did not consume alcohol (AOR = 3.14; 95%CI: 2.24, 4.39) (Fig 8).

## Discussion

Cigarette smoking is one of the leading causes of health risk among all diseases. The WHO Framework Convention on Tobacco Control enlists the monitoring of tobacco use as part of its activities [79]. During the last few years, though, other nicotine delivery products, mainly e-cigarettes, have gained popularity [80,81]. Youngsters, particularly students in whom health-related attitudes are still developing, are most at risk from the use of e-cigarettes [82]. This age group also constitutes a major target of marketing strategies by tobacco and e-cigarette companies [83]. Although several meta-analyses have recently been conducted regarding the use of e-cigarettes in students in Europe and the United States [84], the present study is considered the first global meta-analysis, as per the knowledge of the researchers. Therefore, the objective of this systematic review and meta-analysis was to investigate the overall prevalence of e-cigarette use among students across different parts of the world and the associated factors with this prevalence.

Among the major findings from this analysis was a significant association between gender, the use of convenient cigarettes, and current alcohol consumption with regard to e-cigarette use, as well as the critical review of the usage of e-cigarettes both on the global and regional level. In fact, it was established that the global estimate of the prevalence of e-cigarette smoking was 22.65% [95% CI: 18.32, 26.92]. This represents a higher percentage compared to that in a study from the European region, which had a prevalence of 1^2^4% [85], while it is still less than that contained in another European study, where the prevalence was 43.7% [86]. These differences likely happened due to time gaps between studies and other factors such as sample sizes and socio-demographic characteristics among participants.

Our study also revealed that there was a significant difference in the prevalence of electronic cigarette use by region and economic grouping. Among the WHO regions, lifetime prevalence of e-cigarette use was the highest in the Western Pacific region: 32.13% [95% CI: 18.99, 45.27] and lowest in the Eastern Mediterranean region: 14.43% [95% CI: 12.38, 20.48]. In terms of World Bank regions, the East Asia and Pacific region recorded the highest prevalence of e-cigarette use, at 29.75% (95% CI: 22.21–37.29), whereas the South Asia region recorded the lowest rate of 6.2% (95% CI: 4.09–8.31). Smoking was less common in South Asia mainly due to strong culture and religious practices, more consumption of smokeless tobacco, more unaffordability of cigarettes, and more stringent tobacco control in some countries [87,88]. Apart from that, prevalence of e-cigarette use was more common among high school students 33.62% [95% CI: 25.08–42.16, I^2^ = 55%] than that among university students 20.22% [95%CI: 14.45–25.99, I^2^ = 45%]. Previous studies had shown High education was associated with smoking cessation in the overall adult population, most notably among men.Educational attainment affects health behavior through promoting knowledge, normative influence, reduced stress via work security, and enhanced access to health care. All these pathways together result in decreased smoking among highly educated individuals [89,90].

In the present study, participants’ gender, convenientional cigarettes usage, and currently drinking alcohol were significant global predictors for electronic cigarette use among students. In fact, results showed that male students were three times more likely to engage in electronic cigarette use than female students, supporting studies in Europe [85,86,91,92]. This may be related to the greater tendency of women to seek, obtain, and share information about health, as well as engage in active health behaviors [93]. Prior studies have also found e-cigarette awareness to be substantially higher among males than females, and this disparity in awareness may be further compounded by the greater propensities of males than females to have ever tried or currently use these products. This could be partly explained by the belief of males that the use of an e-cigarette is less dangerous [94].

The results of this meta-analysis revealed that students who used a convenientional cigarette were 5 times more likely to use an electronic cigarette compared to their counterparts. In this case, it would be reasonable to expect to see that youth who are sensation-seeking and/or rebellious would be apt to try both conventional cigarettes and e-cigarettes. Also, students perceive e-cigarettes as less addictive than conventional cigarettes [95].

Those currently using alcohol had four times the odds of using an e-cigarette compared to non-current alcohol users. The use of alcohol in concert with cigarettes is an established trend, and this has been shown in numerous prior studies. Alcohol use disorder has been demonstrated in previous studies to be associated with a greater likelihood for individuals to also use an e-cigarette due to the dopamine reward pathway post both alcohol and nicotine consumption.

The findings of this worldwide systematic review and meta-analysis of e-cigarette prevalence among students emphasize the need for targeted education, regulation, and preventive activity. Education leads to tobacco control. Schools must incorporate effective, evidence-based education on overall e-cigarette health hazards and myths about safety into their curricula, which must be communicated very clearly. Teachers must have the most current knowledge and training to present accurate facts. Peer-led programs and youth participation initiatives can also help enable students to resist marketing and social pressures. Early integration of these initiatives in adolescence can prevent initiation and decrease rates of nicotine addiction among youth.

Regulation-wise, meaningful, enforceable legislation is required to limit youth access to e-cigarettes. Policy makers must enact and enforce age restrictions through credible verification processes, ban flavored products of youth appeal, and require robust health warning labels on packaging. Complete proscriptions on advertisement and marketing, especially on the Internet accessed by youth, are required to reduce the attractiveness of e-cigarettes. Online marketing and cross-border advertising need to be similarly tackled as part of closing loopholes eroding attempts at regulation.

Prevention must be a multi-component, culture-specific response. Media campaigns using relevant messaging and authentic youth influencers can reverse social norms and discourage vaping initiation. Involving the family and community is key to enhancing the extent and impact of prevention activities through targeting of significant social influences. Schools can provide cessation services to students who are already using e-cigarettes. Lastly, ongoing monitoring and data collection are essential to monitor trends, guide policy reform, and evaluate the impact of interventions.

The study has notable strengths, particularly the use of comprehensive worldwide electronic search engines in initiating the research. It set out to determine the prevalence of the use of electronic cigarettes both from an international and regional perspective. In addition, the study established the prevalence of electronic cigarette use both at global and regional levels. However, the study is not without weaknesses. The study has a number of limitations. These make comparisons of results difficult without a meta-analysis conducted on the same population. The lack of data regarding geographic areas like the WHO-defined African region, the World Bank-defined Sub-Saharan, and the World Bank income group-defined low-income limits research. Heterogeneity of the research findings is usually assessed by the I^2^ statistic, but because of the analytical method differences, such as the “Metan” command, it may be not sharply measured. Self-reported data can also be biased due to a social desirability bias in which participants tend to attune their experiences to social expectations, underestimating or overestimating a certain outcome.

## Conclusion

This meta-analysis of 40 studies involving 654,853 students found that 22.65% of students globally reported e-cigarette use. It was most common in the Western Pacific (32.13%), high school students (33.62%), and high-income countries (23.15%). Predictors of importance were male (AOR = 3.22), smoking regular cigarettes (AOR = 5.35), and alcohol use (AOR = 3.14).

These findings portend growing global health anxiety since e-cigarette exposure disproportionately lands on teenagers in high-income and high-prevalence settings. The observation of a robust association with smoking and drinking portends that vaping is a manifestation of a broader pattern of dangerous behaviors with potential to lead to long-term nicotine addiction and heightened susceptibility to follow-on tobacco consumption.

There is a need to implement focused, school-based education in high-risk groups such as high school students and young males. Policymakers, especially in areas of high prevalence, need to strengthen regulation of youth availability, flavored products, and promotion. Multimodal prevention strategies that include education, family engagement, and Internet-based engagement with increased global surveillance are paramount to halt this growing trend and protect young health.

## Supporting information

S1 File Prisma checklist. (DOCX)

S2 File Methodological quality assessment of included studies using Joanna Brigg’s Institute quality appraisal criteria scale (JBI). The eight-item questions assessing inclusion criteria, study setting and participant, exposure measurement, objectives, confounder, statically analysis, outcome measurement, and dealing confounder were used.
(DOCX)

S3 File Dataset. (XLSX)
