# Supplementary material for: Global prevalence of E-cigarette use among students: Systematic review and meta-analysis
Source: PLoS One. 2025 Dec 1;20(12):e0332160. doi: 10.1371/journal.pone.0332160 (PMC12668485; doi:10.1371/journal.pone.0332160)
Supplement: S2 File — The eight-item questions assessing inclusion criteria, study setting and participant, exposure measurement, objectives, confounder, statically analysis, outcome measurement, and dealing confounder were used. (DOCX) [file pone.0332160.s002.docx]

Additional file2. Quality assessment for the included Studies

| Item | Clearly defined inclusion | Describe study setting and participant | Valid and reliable exposure measurement | Objective and standard criteria for measurement | Identified confounder | Strategies to deal with confounders | Valid and reliable outcome measurement | Appropriate statically analysis | No of ‘yes’s ‘ |
| --- | --- | --- | --- | --- | --- | --- | --- | --- | --- |
| Duplaga M and Grysztar M/2022 | Yes | Yes | No | Yes | Yes | No | Yes | Yes | 6/8=75 |
| Leung J. et.al/2023 | Yes | Yes | Yes | Yes | No | No | Yes | Yes | 6/8=75 |
| Serra, C/2021 | Yes | Yes | No | Yes | Yes | No | Yes | Yes | 6/8=75 |
| Janik-Koncewicz K. et.al/2020 | Yes | Yes | No | Yes | Yes | Yes | Yes | Yes | 7/8=87.5 |
| Chen J. et.al/2019 | Yes | Yes | No | Yes | Yes | Yes | Yes | Yes | 7/8=87.5 |
| Soteriades, S et.al/2020 | Yes | Yes | Yes | Yes | Yes | No | Yes | Yes | 7/8=87.5 |
| Huang LL. et.al/2016 | Yes | Yes | No | Yes | Yes | Yes | Yes | Yes | 7/8=87.5 |
| Bigwanto et al/2019 | Yes | Yes | Yes | Yes | No | No | Yes | Yes | 6/8=75 |
| E. Westling et al/2017 | Yes | Yes | No | Yes | Yes | No | Yes | Yes | 6/8=75 |
| S. Azagba, et al/2019 | Yes | Yes | Yes | Yes | Yes | No | Yes | Yes | 7/8=87.5 |
| Qanash, et al/2018 | Yes | Yes | Yes | Yes | No | No | Yes | Yes | 6/8=75 |
| Alzahrani T/2023 | Yes | Yes | No | Yes | Yes | Yes | Yes | Yes | 7/8=87.5 |
| Tavolacci M-P/2016 | Yes | Yes | No | Yes | Yes | No | Yes | Yes | 6/8=75 |
| A.M. Franks et al/2017 | Yes | Yes | Yes | Yes | No | No | Yes | Yes | 6/8=75 |
| E.L. Sutfin et al/2013 | Yes | Yes | No | Yes | Yes | No | Yes | Yes | 6/8=75 |
| Song et al/2023 | Yes | Yes | No | Yes | Yes | Yes | Yes | Yes | 7/8=87.5 |
| Babjakova, J/2020 | Yes | Yes | No | Yes | Yes | Yes | Yes | Yes | 7/8=87.5 |
| Alzalabani and Eltaher/2020 | Yes | Yes | Yes | Yes | Yes | No | Yes | Yes | 7/8=87.5 |
| Iqbal et al/2018 | Yes | Yes | No | Yes | Yes | Yes | Yes | Yes | 7/8=87.5 |
| Irena Ilic et.al /2019 | Yes | Yes | Yes | Yes | No | No | Yes | Yes | 6/8=75 |
| Canzan et al./2019 | Yes | Yes | No | Yes | Yes | No | Yes | Yes | 6/8=75 |
| Ghanim M/2024 | Yes | Yes | Yes | Yes | Yes | No | Yes | Yes | 7/8=87.5 |
| Puteh SE et.al/2018 | Yes | Yes | Yes | Yes | No | No | Yes | Yes | 6/8=75 |
| Nazzal Z, et al/2024 | Yes | Yes | No | Yes | Yes | Yes | Yes | Yes | 7/8=87.5 |
| Le Thi Thanh Huong et al/2022 | Yes | Yes | No | Yes | Yes | No | Yes | Yes | 6/8=75 |
| Al-Sawalha et al/2021 | Yes | Yes | Yes | Yes | No | No | Yes | Yes | 6/8=75 |
| D. R. Kenne et al/2015 | Yes | Yes | No | Yes | Yes | No | Yes | Yes | 6/8=75 |
| Balogh et al/2018 | Yes | Yes | No | Yes | Yes | Yes | Yes | Yes | 7/8=87.5 |
| Habib, et al/2020 | Yes | Yes | No | Yes | Yes | Yes | Yes | Yes | 7/8=87.5 |
| Oliveira WJC/2017 | Yes | Yes | Yes | Yes | Yes | No | Yes | Yes | 7/8=87.5 |
| Kurdi et al/2021 | Yes | Yes | No | Yes | Yes | Yes | Yes | Yes | 7/8=87.5 |
| Wamamili B, et al/2020 | Yes | Yes | Yes | Yes | No | No | Yes | Yes | 6/8=75 |
| Kinouani S/2017 | Yes | Yes | No | Yes | Yes | No | Yes | Yes | 6/8=75 |
| Jane Ling et al/2022 | Yes | Yes | Yes | Yes | Yes | No | Yes | Yes | 7/8=87.5 |
| Susi Ari Kristina et al/2020 | Yes | Yes | Yes | Yes | No | No | Yes | Yes | 6/8=75 |
| Ivan Cherrez-Ojeda/2024 | Yes | Yes | No | Yes | Yes | Yes | Yes | Yes | 7/8=87.5 |
| Annie Montreuil et.al/2017 | Yes | Yes | No | Yes | Yes | No | Yes | Yes | 6/8=75 |
| Muhammad Ahmed S/ 2024 | Yes | Yes | Yes | Yes | No | No | Yes | Yes | 6/8=75 |
| Wichaidit W et.al/2023 | Yes | Yes | No | Yes | Yes | No | Yes | Yes | 6/8=75 |
| Andrew K et.al/2015 | Yes | Yes | No | Yes | Yes | No | Yes | Yes | 6/8=75 |
